# Trade-offs between acquired and innate immune defenses in humans

**DOI:** 10.1093/emph/eov033

**Published:** 2016-01-06

**Authors:** Thomas W. McDade, Alexander V. Georgiev, Christopher W. Kuzawa

**Affiliations:** ^1^Department of Anthropology, Institute for Policy Research, Northwestern University, Evanston, IL 60208; ^2^Child and Brain Development Program, Canadian Institute for Advanced Research, Toronto, Ontario M5G 1Z8, Canada

**Keywords:** inflammation, immune function, ecological immunology, developmental origins of health and disease

## Abstract

Immune defenses provide resistance against infectious disease that is critical to survival. But immune defenses are costly, and limited resources allocated to immunity are not available for other physiological or developmental processes. We propose a framework for explaining variation in patterns of investment in two important subsystems of anti-pathogen defense: innate (non-specific) and acquired (specific) immunity. The developmental costs of acquired immunity are high, but the costs of maintenance and activation are relatively low. Innate immunity imposes lower upfront developmental costs, but higher operating costs. Innate defenses are mobilized quickly and are effective against novel pathogens. Acquired responses are less effective against novel exposures, but more effective against secondary exposures due to immunological memory. Based on their distinct profiles of costs and effectiveness, we propose that the balance of investment in innate versus acquired immunity is variable, and that this balance is optimized in response to local ecological conditions early in development. Nutritional abundance, high pathogen exposure and low signals of extrinsic mortality risk during sensitive periods of immune development should all favor relatively higher levels of investment in acquired immunity. Undernutrition, low pathogen exposure, and high mortality risk should favor innate immune defenses. The hypothesis provides a framework for organizing prior empirical research on the impact of developmental environments on innate and acquired immunity, and suggests promising directions for future research in human ecological immunology.

## INTRODUCTION

Recent clinical and epidemiological research has implicated chronic inflammation in the etiology of a wide range of chronic degenerative diseases, motivating intense interest in the proximate factors that influence the regulation of inflammation [1–4]. Yet the critical role inflammation plays as part of innate immune defenses has been known for nearly 2000 years [5], and it is only recently that inflammation has been pathologized and its importance to survival discounted [6]. There is tremendous variation in levels of inflammation, within and across populations, yet scientific understandings of the causes and consequences of this variation are lacking.

Similarly, a longstanding tradition of research in immunology and public health has sought to understand ways to enhance acquired immune defenses to reduce burdens of morbidity and mortality associated with infectious diseases, particularly among the elderly, and among children in lower income nations [7, 8]. Studying the response to vaccination, for example, has yielded insights into the mechanisms of immunological memory and strategies for bolstering effectiveness of vaccination programs [9]. However, the typical biomedical approach fails to recognize that immune defenses are costly, and that maximizing investments in immunity and long-term survival may not always serve an organism’s fitness goals.

In this article, we propose a framework for explaining variation in patterns of investment in two important subsystems of anti-pathogen defense: innate and acquired immunity. We draw on concepts from evolutionary life history theory, and build on a foundation of theoretical and empirical research in non-human vertebrate ecoimmunology. Our focus is on humans, and we consider the relative costs and effectiveness of each subsystem of defense, and propose that the balance of investment in innate versus acquired immunity will be shaped by three ecological factors during sensitive periods of immune development: (i) the availability of nutritional resources, (ii) the intensity of pathogen exposure and (iii) signals of extrinsic mortality risk. We also hypothesize that, as a result of this phenotypic calibration, the relative investment in innate versus acquired immunity will co-vary with life-history traits related to reproductive scheduling (e.g. tempo of sexual maturation, age at first sexual intercourse and age at first reproduction), which are also responsive to ecological signals reflecting nutritional condition and mortality risk.

These hypotheses organize prior empirical research on the impact of developmental environments on aspects of human immunity, and suggest promising directions for future research. They also build on our prior theoretical and empirical work in human ecological immunology, which has also focused on life history trade-offs and the costs of immunity [6, 10, 11]. However, to the best of our knowledge, this article is the first to focus specifically on elaborating a framework for predicting trade-offs within the human immune system, across subsystems of innate and acquired defenses.

## LIFE HISTORY THEORY AND ECOLOGICAL IMMUNOLOGY

A life history perspective bridges ecology and evolutionary biology, and provides a framework for understanding variation in reproductive and developmental strategies, both within and across species [12, 13]. A key assumption of life history theory is that resources are limited, and that according to the ‘allocation rule’, resources invested in one area are not available for use in another [14]. Trade-offs are therefore inevitable, and organisms strive to allocate limited resources across the life cycle, within a given ecological context, in ways that maximize reproductive fitness.

A second assumption is that extrinsic mortality risk—defined as the likelihood of death from exogenous sources in the local environment—is an important driver of variation in mammalian life history strategies [15]. Because the reproductive benefits of investing in a durable soma are deferred, the utility of allocating limited resources to maintenance activities is dependent upon the likelihood of future survival, which is a function of unavoidable risks faced by members of a population or species. Species living in high risk environments are less likely to live into the future, and are thus predicted to invest more of their limited resources into processes that facilitate current reproduction, with relatively fewer resources allocated to maintain a ‘disposable’ soma [16]. As a result, ‘fast’ life histories are typically associated with shorter life spans, earlier reproduction and the production of many offspring with lower parental investment. Conversely, when extrinsic mortality risk is low, greater investment in maintenance and the soma is predicted, which facilitates a ‘slow’ life history characterized by longer life span and reduced expenditure on growth or reproduction, and the production of fewer offspring with a higher level of parental investment and survival [17].

Resources and risks in the local environment are therefore conceptualized as important determinants of developmental and reproductive strategies. The optimal resolution to a given trade-off, associated with the allocation of limited resources, can be genetically encoded as the result of natural selection in a stable environment, or developmentally mediated as the result of facultative adaptation during an individual’s lifetime. The former process is often invoked to explain between-species variation in life history traits. The latter process is often used to explain variation within the human species, under the assumption that humans have the ability to respond adaptively to a wide range of ecological pressures within a ‘reaction norm’ [14, 18].

Immunity represents a primary physiological system that plays an essential role in survival. It is therefore a major component of maintenance effort, investments in which are likely calibrated to resource availability and risks in the local environment. Research in ecological immunology, drawing on this evolutionary life history framework, recognizes that investments in immune defenses are costly, and that allocations of limited resources to immunity are not available for other physiological or developmental processes [19–22]. The optimal allocation of resources to subsystems of immunity, therefore, may vary depending on the exigencies of the local ecology [23]. Multiple studies, across a wide range of species and ecological settings, have documented significant trade-offs between immune defenses and investments in growth and reproduction, as well as trade-offs between investments in innate versus acquired immunity [19, 20, 24–32]. Recent studies with humans have further underscored the value of a life history approach to understanding variation in immune development and function [10, 11, 33, 34], which we explore in detail below.

## HUMAN IMMUNE DEFENSES: FUNCTIONS AND COSTS

### Innate versus acquired immunity

As with all vertebrates, the human immune system is comprised of several interdependent subsystems of activity and defense [35]. There exists a fundamental distinction between innate, or non-specific defenses, and acquired, or specific immune defenses. Innate immune defenses provide resistance to disease without recognizing specific antigens, and include anatomical barriers such as skin and mucosal membranes, anti-microbial soluble proteins (e.g. complement, lysozyme), natural killer cells and phagocytic cells (macrophages, neutrophils, dendritic cells). The inflammatory response—involving acute phase proteins and the recruitment of phagocytic cells to the site of injury or infection—is an important aspect of innate immune defenses against a wide range of pathogens.

In contrast, defining features of acquired immunity include the ability to recognize and target antigens with exquisite precision (specificity), and more rapid and effective responses to antigens upon secondary exposure (memory). T and B lymphocytes are the central cellular mediators of specific immunity, and an exposure-driven, Darwinian process of clonal selection is necessary for the development of acquired immunity. Mature T and B lymphocytes express unique antigenic receptors, the functional form of which results from the random rearrangement of mini-gene segments, imprecise joining of nucleotide sequences, and random combinations of peptide chains. Although the human genome contains <25 000 genes, this developmental process can produce well over 100 million different antigen-binding specificities [35, 36]. The generation of large numbers of random variants increases the likelihood that any one will bind, or ‘recognize’, an antigen, representing an elegant response to the challenge posed by infectious microbes: relatively short intergenerational intervals and high mutation rates that increase the potential for the evolution of resistance [37].

With a diverse pool of T and B lymphocytes in place, antigenic exposures drive the maturation of acquired immune defenses by binding to, and activating, specific lymphocytes with matching receptors. Selected lymphocytes undergo mitosis, passing on their genes to subsequent generations of daughter cells that share the same antigenic specificity. These cell lines activate effector functions that control and remove the antigen, and they differentiate into long-lived memory cells that initiate stronger and more rapid responses upon subsequent encounters with the same antigen [35].

Significantly, the development of acquired immunity is a time-dependent, exposure-driven process during which the individual ‘learns’ about the local infectious disease ecology. In contrast, innate immunity relies on pattern recognition receptors against highly conserved elements of infectious agents, encoded in the germ line, with no developmental process leading to more finely tuned responses. The innate immune response to a particular pathogen is the same upon first exposure as it is following all subsequent exposures (i.e. no memory), and all innate immune cells (within a particular subtype) are equally capable of responding to a particular pathogen (i.e. no specificity). These distinctions shape the relative costs and benefits of innate and acquired immunity, with implications for trade-offs in immune investments.

### Costs of immunity

The costs of immunity are substantial, variable across subsystems of defense, and shaped by local ecological conditions. Relatively few studies have attempted to quantify the costs of immunity in humans, although there are many observational and experimental studies with rodents, birds and insects that document substantial energetic, reproductive and survival costs of immune activation [38]. Prior work on humans has focused almost exclusively on the overall costs of immunity, and the potential for trade-offs with systems or processes related to growth or reproduction [11, 38]. Here, we build on prior work in ecological immunology [29, 30] to distinguish the costs of innate versus acquired immunity, and we consider relative costs of the development, maintenance and activation of innate versus acquired defenses (Table 1).

**Table 1.** Costs of innate and acquired immune defenses

	**Costs**				**Effectiveness**	
	Developmental	Maintenance	Activation	Collateral damage	Novel/primary exposures	Secondary/ future exposures
**Innate immunity**	low	medium	high	medium	good	good
**Acquired immunity**	very high	low	low	low	poor	excellent

Source: adapted from Klasing (1999).

The development of acquired immunity requires energy and biochemical substrates (i.e. amino acids, lipids) to fuel the generation of a large pool of T and B lymphocytes with diverse antigen binding specificities. In addition, investments in lymphoid tissues are needed to support lymphocyte development and function. The thymus—central to T lymphocyte maturation and differentiation—imposes rigorous selection procedures to eliminate self-reactive cell lines, which can emerge from the stochastic processes used to generate antigen-binding receptors. It has been estimated that over 95% of T lymphocytes are destroyed in the thymus prior to their release into circulation, making this an expensive and inefficient developmental process [39]. In addition, the volume of functional thymic tissue is largest in infancy, and the thymus gradually declines to less than half its original size by adulthood [40], thereby imposing disproportionately higher costs early in life, during critical periods of acquired immune development.

An additional developmental cost of acquired immunity is time: As noted earlier, clonal selection and the establishment of immunological memory requires exposure to antigens in the local environment. Therefore, the benefits of more rapid and effective secondary responses are only realized with subsequent exposures to the same pathogens, while acquired immune responses are slower and less effective upon initial exposure. The time- and exposure-dependent nature of acquired immune development therefore imposes a window of enhanced vulnerability to infectious disease early in life. In other words, investing in acquired immunity requires the payment of upfront costs in anticipation of deferred future benefits.

The development of innate immunity also requires energy and biochemical substrates, but these costs are lower since the developmental process is relatively gradual and efficient. Innate immune cells express shared sets of pattern recognition receptors, with no need for the over-production and selective retention of functional cell lines.

The maintenance costs of both acquired and innate immunity are hypothesized to be low, and are defined primarily by the allocation of energetic and biochemical resources to replace cells (e.g. lymphocytes, phagocytic cells) and soluble proteins (e.g. immunoglobulins, complement) with limited half-lives [29]. Lymphocytes are present in circulation in smaller numbers, and turn over less frequently, than phagocytic cells [35], suggesting that the maintenance costs of acquired immunity are lower than for innate immunity.

The overall costs of activating immune defenses are substantially higher, with resources required to fuel the acute phase response, proliferation of leukocytes, and the production and release of immunoglobulins, cytokines, and other effector mechanisms. In humans, fever generates a 13% increase in metabolic rate for each degree Celsius increase in body temperature [41], and in birds, immune activation has been associated with a 29% increase in metabolic rate [42]. A recent, naturalistic study of respiratory infection documented an 8% increase in resting metabolic rate among young men during illness [38]. In addition to the direct metabolic costs, immune responses can lead to anorexia and/or diarrhea, which can disrupt normal processes of nutrient intake, digestion and absorption [43,44], further compromising nutritional resources. Collateral damage and loss of function (short and long term), as well suppression of reproductive function [38], are also potential costs of immune activation.

It is difficult to isolate costs associated with the activation of innate versus acquired defenses, since in humans the typical response involves both arms of immunity. However, it can be inferred that acquired immune defenses—particularly secondary, memory responses—are likely to consume fewer nutritional resources on a per-response basis, and are also less likely to inflict collateral damage [28, 29]. Innate responses are potentially more damaging due to their lack of specificity, and require more resources due to the exponential increase in protein production associated with the systemic acute phase response, along with the high metabolic costs of fever. For example, a classic experiment that involved the injection of endotoxin in adults has been associated with up to 40% increase in basal metabolic rate, with a mean response of ∼20% [45]. The costs of this response can be attributed to innate immunity, since endotoxin is an inactivated bacterial product that rapidly mobilizes the acute phase response, including fever, through the activation of highly conserved pattern recognition receptors.

Vaccine studies provide some insight into the resource costs of acquired immune activation, particularly primary versus secondary responses. For example, infants receiving a vaccine against bacterial meningitis mounted a primary response which included, on average, the production of 11 memory B cells per 10^6^ total lymphocytes and 19.8 μg/ml IgG antibodies, both specific to the antigen in the vaccine [46]. More than 7 months later, after being challenged with the same vaccine, the infants mounted a secondary response that resulted in the production of 40 per 10^6^ memory B cells and 46.2 μg/ml IgG antibodies against the antigen. A rapid and robust response is a defining feature of immunological memory, and in this case the production of key effectors—antigen-specific antibodies and B lymphocytes—was 2.3–3.6 times greater following secondary exposure.

However, in absolute terms, the resources required to fuel this response are quite modest. The primary response resulted in the production of 19.8 μg/ml specific IgG antibody, which translates into ∼11.6 mg of total protein production, after adjusting for body size and plasma volume (These calculations are based on the following assumptions [47, 48]: body weight = 10 kg; plasma volume = 58.7 ml/kg; total IgG =3375 mg; total lymphocytes = 2.5 × 10^9^; total serum protein= 41 g.). The secondary response resulted in 27.1 mg total IgG protein production, an additional cost of 15.5 mg in comparison with the primary response. As a point of reference, the average amount of total IgG in infants is 3375 mg, and the total amount of serum protein is 41 g. Protein production specific to the secondary antibody response therefore represents only 0.46% of the total amount of IgG, and only 0.038% of total serum protein. Furthermore, it has been estimated that a marginally nourished child synthesizes ∼3.5 g of protein per kg of body weight each day, and that the rate of turnover increases to 6.0 g in the presence of infection [49]. For a 10 kg infant, 15.5 mg of protein therefore represents only 0.043% of the total daily protein budget, and only 0.026% of total protein production during infection.

Consideration of B lymphocyte proliferation following secondary exposure paints a similar picture. The increase in memory B lymphocytes from 11 to 40 per 10^6^ lymphocytes translates into a total of 72 500 additional cells in circulation, which represents only 0.0029% of the total lymphocyte pool. In addition, it has been estimated that the dry mass of stimulated lymphocytes is ∼400 pg [50]. More than half this mass will be protein, and even if it is assumed that the entire mass is protein, the total protein cost of these additional 72 500 lymphocytes is only 0.029 mg, or 0.00048% of total daily protein production during infection.

Obviously, these calculations do not provide a complete accounting of the resources required to activate acquired immune defenses, but they underscore the point that the absolute costs of response can be quite low, even when the magnitude of a secondary response is large in comparison with the primary response. For the sake of our argument it is not necessary to have a precise enumeration of the actual costs of innate versus acquired immune defenses: The relative costs of development, maintenance, and activation, and the relative effectiveness against primary versus secondary exposures, are the key factors that likely structure trade-offs in investments across ecological settings.

In sum, the developmental costs of acquired immunity are exceptionally high, but the costs of maintenance and activation are relatively low. Innate immunity, in contrast, imposes lower upfront developmental costs, but higher operating costs. Innate defenses are mobilized quickly and provide a critical line of defense against novel pathogens, and responses do not change with subsequent exposures. Acquired responses are comparatively slow and relatively ineffective against novel exposures, but they are more rapid, more effective and probably less costly on a per response basis than innate defenses following future, secondary exposures due to immunological memory.

## TRADE-OFFS BETWEEN INNATE AND ACQUIRED IMMUNITY IN NON-HUMAN VERTEBRATES

The benefits and costs of immune defenses, as outlined above, are broadly similar across all vertebrates, but species differ considerably in levels of investment in innate and acquired defenses. The ‘pace-of-life’ hypothesis proposes that species on the slower end of the life history continuum (i.e. slower growth, delayed reproduction, higher levels of parental investment per offspring, longer life spans) should invest more in developmentally costly immune defenses like acquired immunity [27]. Conversely, species characterized by a fast life history are less likely to survive into the future to reap the deferred benefits of acquired immunity, and should therefore be biased toward investments in innate immunity, with lower upfront costs and generally good protection against a wide range of pathogens.

Predictions from the pace-of-life hypothesis have received mixed support. In a large comparative study of 70 species of neotropical birds [51], length of egg incubation (an indicator of pace of life) was positively associated with natural antibody levels (used here as a measure of acquired defenses), suggesting that a slower pace of life predicts greater investment in acquired immunity. Conversely, complement (a part of the innate response) was weakly but positively correlated with clutch size, suggesting that species emphasizing quantity, rather than quality of offspring (‘fast’ pace-of-life), invest more in innate immunity.

A comparative study of three rodent species, across different genera at distinct locations along the slow-fast life history continuum, similarly supported the pace-of-life hypothesis [52]. The species with the fastest life history demonstrated the highest level of investment in innate immunity (bacteria killing activity), and the lowest level of acquired immunity (antibody response to a foreign agent). Conversely, the species at the slow end of the life history continuum had the highest level of acquired immunity and the lowest level of innate immunity. However, a similarly designed study of six other rodent species from the same genus (*Peromyscus*) involving captive-bred, disease-free individuals did not support the predictions of the pace-of-life hypothesis [53]. Still, the results showed that there were species-level trade-offs in immune defenses such that individuals from a given species had either strong innate or acquired responses but not both [53]. It is important to note that using controlled laboratory conditions, under which the subjects of this study were bred and tested, effectively rules out any confounding effects due to environmental variation in pathogen exposure, allowing the analyses to focus on the effects of the species-typical life history patterns. The fact that the predictions of the pace-of-life hypothesis were not supported suggests that the environment may have a key role in modulating immune phenotype.

The ‘antigen-exposure’ hypothesis foregrounds ecological variation in pathogen pressure as a key determinant of variation in immunity. It predicts that in high pathogen environments, overall investment in immune function will be greater, and/or that acquired immunity will be prioritized over innate immunity if the costs of increasing investment in all aspects of immune function are too high [54–56]. In support of this hypothesis, finches living on larger islands in the Galapagos with a greater prevalence of parasites had higher levels of acquired immunity [54]. Conversely, birds living on smaller islands showed greater levels of innate immune response. However, no trade-offs between innate and acquired immune defenses were detected at the individual level [54].

A shortcoming of prior research is that few studies have simultaneously considered multiple potential determinants of variation in immune allocation strategies. An exception is a recent study of 23 populations of 12 different lark species, in which several measures of innate immune function showed more consistent relationships with environmental proxies of pathogen exposure, rather than with proxies of pace of life [57]. Specifically, larks living in mesic, more pathogen-rich environments invested in higher levels of innate immune defenses on two out of four measures compared with those living in arid, lower pathogen environments. Of the four immune measures, only one correlated with a measure of the pace-of-life (number of eggs per clutch): species laying more eggs had higher agglutination titers, indicating greater innate immune investment among species with a faster pace of life. The authors interpreted these findings as contradicting the pace-of-life hypothesis, and supporting the antigen exposure hypothesis. But an issue with such an interpretation is that only measures of innate immunity were used and thus a more detailed examination regarding within-immune system trade-offs is not possible. The findings nevertheless suggest that environmental factors might have greater impact on variation in investment towards immune defenses than species-typical pace-of-life traits, at least as far as innate immunity is concerned. Similarly, empirical analyses demonstrating inconsistent patterns of co-variation in immune, endocrine and metabolic traits among individuals and between subspecies argue against the existence of a single life-history/physiology axis [58].

An additional limitation is that prior research has not given adequate attention to nutrient access as a potential determinant of immunophenotypes. Nutritional resources are needed to fuel immune development and function, and limited resources influence the nature and severity of trade-offs within the immune system, and with other physiological processes [23, 59–61]. For example, in a study of tree swallow nestlings, multiple markers of innate and acquired immunity were assessed across habitats with low and high food quality [62]. There was some evidence for trade-offs between innate and acquired immune measures in poor quality habitats, but also a significant amount of inter-site and inter-annual variation that did not align with the predictions of the study.

In sum, animal studies in ecoimmunology have focused on the location of species along the slow-fast continuum, and pathogen exposure in the local environment, as the main factors shaping the balance of investment in innate and acquired immune defenses. Few studies have considered nutritional resources, and fewer still have simultaneously evaluated multiple predictors of variation in immunity. The extent to which these frameworks will translate to humans is not known, but they provide a reasonable empirical foundation upon which to construct an integrated theoretical framework that will advance our understanding of human immunophenotypes.

## HYPOTHESES: INVESTMENTS IN INNATE AND ACQUIRED IMMUNITY IN HUMANS

**Figure 1.**
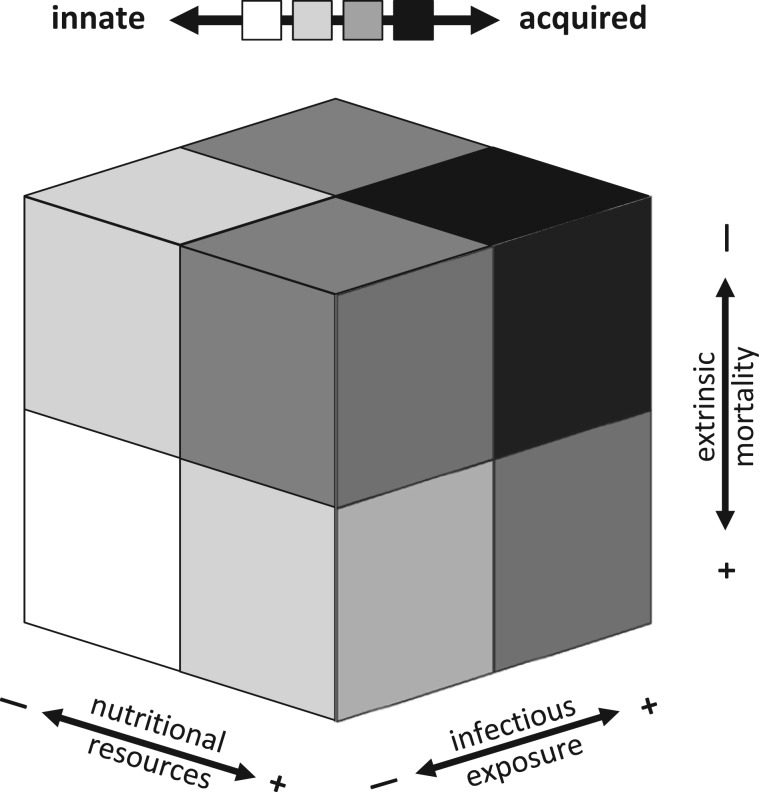
Higher levels of nutritional resources, more intense pathogen exposure, and lower extrinsic mortality risk should promote higher levels of investment in acquired immune defenses (darker gray to black). Lower levels of nutritional resources and infectious exposures, and higher extrinsic mortality, will promote more investment in innate immunity (lighter gray to white). Absent from view is the condition with low nutritional resources, high infectious exposure, and high extrinsic mortality, which should bias investment toward innate immunity (lighter gray)

Based on the relative costs and benefits of innate and acquired immune defenses, we propose that local ecological conditions early in development influence patterns of investment across these subsystems of defense. Humans reside at the extreme end of the ‘live-fast-and-die-young’ continuum, with low extrinsic mortality, long lifespans, slow growth, and intense parental investment. From this perspective, the pace-of-life hypothesis predicts a universally high level of investment in acquired immunity for the human species [29, 63]. However, even among humans, there are large reaction norms in reproductive and life-history strategies, with considerable within-species variability along the slow–fast continuum [64]. It is also clear that there is tremendous within-species variation in immune parameters, and that developmental plasticity and ecological sensitivity are defining features of the human immune system [10]. Our framework attempts to explain this variation, and proposes that the balance of investment in innate versus acquired immune defenses will be shaped by (i) nutrition (available resources), (ii) infectious exposure (ecological demands on the system) and (iii) extrinsic mortality risk (probability of future survival, not contingent on allocations to somatic or reproductive effort) (Fig. 1).

### Nutritional resources

Energy and biochemical substrates are necessary for the development, maintenance and activation of immune defenses. In the absence of adequate nutrition, the development and function of the immune system may be constrained, with subsystems of defense that require more resources disproportionately affected. The availability of adequate nutritional resources may also function as a signal that forecasts future resource availability [65, 66] thereby guiding allocations of resources to physiological systems during sensitive periods of development [67, 68]. In other words, if nutritional resources are limited in the local environment, then investments should be biased toward systems that require fewer resources, both in the short and long term.

Based on the high energetic and biochemical substrate costs of acquired immunity during development, we hypothesize that nutritionally marginal prenatal and early postnatal environments will favor lower levels of investment in acquired immunity. Although humans like all mammals face particularly high energetic stress during infancy and childhood [10, 69], the energetic demands of brain development may place unique constraints on energetic expenditures and related trade-offs during human childhood. In humans, the brain reaches its peak expenditure at 4–5 years of age, when it accounts for 66% of resting expenditure [70]. Growth rate and brain expenditure are tightly, inversely related during childhood, pointing to energetic trade-offs between these competing functions. We speculate that the unusually strong energetic trade-offs of human infancy and childhood may, under nutritionally marginal settings, have a significant impact on the ability to invest in costly acquired immune defenses, biasing expenditures toward long-term investments in innate immunity. We acknowledge that marginal nutrition might be expected to favor acquired immunity in the long run, given the lower maintenance and per-response activation costs. However, the high developmental costs serve as a barrier that must be overcome before this phenotype can be achieved.

We also hypothesize that the effects of nutritional environments on immunity may differ across the life course. Nutritional environments early in life have a disproportionate impact by shaping the allocation of resources across subsystems and establishing regulatory set points during sensitive periods of immune development. The impact of nutrition in adulthood operates through this scaffolding. For example, according to our framework, marginal nutrition early in development should reduce investment in acquired immunity and enhance investment in innate immunity. The opposite pattern should emerge in richer nutritional environments. We suggest that the impact of nutritional availability in adulthood may differ based on these early environments: in environments of caloric excess that are driving rising rates of overweight and obesity globally [71], energetic resources will be readily available to fuel acquired as well as innate immune responses. However, among adults whose immune systems developed in the context of marginal nutrition, the window of opportunity for calibrating investments in immunity will have been long closed, and resource abundance in adulthood will not be sufficient to overcome reduced allocations to acquired immunity during sensitive periods of immune development. In these situations, one might expect nutritional abundance to lead to exaggerated innate and inflammatory responses, deriving from a phenotype that is biased toward innate immunity and a nutritional environment that provides abundant resources to fuel innate responses despite their high activation costs.

### Pathogen exposure

On a mechanical level, antigen exposures drive the selection of specific cell lines, which define the mature pool of T and B lymphocytes. Different antigenic exposures across environments result in different lymphocyte pools. In an environment without any threat of microbial exposure and infection, investing in immune defenses would be unnecessary, and limited resources would be better allocated to other processes that promote growth and reproduction. But pathogens are ubiquitous, and they pose substantial threats to growth, reproduction and survival. Therefore, the costs of immunity must be paid, and as the intensity of pathogen exposure increases, immune allocations gain priority over competing life history demands [28]. These allocations may result from mechanisms like antigen-driven clonal selection, and/or the establishment of regulatory set points and the expansion of immune-related tissues that reflect relative levels of somatic investment in aspects of immunity.

We hypothesize that all else equal, higher pathogen load will lead to higher levels of investment in acquired immunity. For the sake of argument we lump all types of pathogens together, but we acknowledge that distinct categories of pathogens (i.e. viruses, bacteria, fungi, helminths), and specific pathogens within each category, have direct implications for the pattern of immune response. In a long-lived species like humans, the probability of repeat exposures to recognized pathogens is high, which enhances the value of the rapid, efficient and relatively low-cost responses driven by immunological memory. With higher pathogen load, the long-term benefits of acquired immunity are more likely to offset the high upfront developmental costs. Similarly, with immunological memory the relative proportion of secondary pathogenic exposures increases over time, thereby reducing the frequency of activation of more costly innate defenses. With low levels of pathogen exposure, the chances of repeat encounters are likely reduced, while the proportion of novel exposures is relatively high. Under this scenario, the relative effectiveness of innate immune defenses, and the low upfront developmental costs, may bias investments toward innate immunity.

Pathogenicity in the local environment may also serve as a signal of mortality risk that influences life history scheduling and immune allocation strategies. For example, among marine gastropods, individuals from a population with higher prevalence of trematodes have been shown to reach maturity earlier than individuals in less-parasitized populations [72]. Similarly, in a historic human population, men who were exposed to a highly infectious pre- and early postnatal environment reproduced earlier than men whose early development fell outside local peaks of high infectious mortality [73]. From this perspective, a high pathogen environment would be predicted to promote greater investment in innate immunity, which provides enhanced protection in the short-term, while discounting the future benefits accrued from investments in acquired immunity.

However, we argue that pathogen load is best interpreted as contributing to intrinsic mortality risk in humans, rather than indicating risk of death from extrinsic sources. Extrinsic mortality risk is by definition unavoidable, imposed by the local ecology and not modifiable by the organism. Intrinsic mortality risk, by contrast, is sensitive to allocations to somatic and reproductive effort, and investments in defensive structures and behaviors [13]. Pathogen exposures lead to infections that have consequences for productivity and survival, but these consequences are a product of the performance of immune defenses. More effective immune defenses reduce the impact of infections; therefore, pathogenicity does not represent an unavoidable, exogenous aspect of the environment with respect to mortality risk. From this perspective, higher levels of exposure to infectious agents in the local environment may signal pressure to invest in the more effective defenses afforded by acquired immunity.

### Extrinsic mortality risk

As noted earlier, extrinsic risk of mortality is recognized as a key driver of mammalian life history variation [13, 15, 64], with implications for immune development and function. In humans, this logic has been applied to explain within-species variation in reproductive scheduling in relation to early life stressors [74]. For example, children exposed to cues of high extrinsic mortality (e.g. death of a sibling) or low parental investment (e.g. parental absence) have been shown to mature earlier in some populations [64, 75, 76] and also to start reproducing at a younger age [77–83].

Building from these findings, we hypothesize that measures of childhood environmental harshness or unpredictability will lead to reduced investments in acquired immune defenses, and relatively higher levels of investment in innate immunity. It takes time to develop acquired immune defenses, and the future benefits of lower cost, more effective memory responses are discounted when the probability of living into the future is low. In the context of elevated mortality risk, therefore, investments in innate immunity would be expected to provide good protection against infectious disease with minimal upfront costs, and resources diverted from the development of acquired immunity could be invested in processes that accelerate growth and/or reproduction.

As represented in Figure 1, our model proposes that the overall effects of nutritional resources, infectious exposures, and extrinsic mortality risk are additive. Investment in acquired immunity will be highest when environments early in development are characterized by nutritional surpluses, higher levels of infectious exposure, and lower mortality risk. The bias toward innate immunity will be highest in the context of marginal nutrition, low levels of infectious exposure, and high extrinsic mortality. Intermediate levels of exposure, or high levels of exposure in one domain and low levels in another, will lead to relatively equal investment in innate versus acquired immunity. However, we acknowledge that interactions across the domains are possible. For example, very low levels of nutritional resources may serve as a constraint on the development of acquired immunity in high pathogen load environments that would otherwise favor higher levels of investment in acquired defenses. Additional research is needed to determine whether permissive effects or other interactions best characterize these patterns of association.

### Life history correlation

Although our framework emphasizes the ecological factors that influence the balance of innate and acquired immunity, it implies a set of hypotheses regarding correlations between immune investments and other life history traits. Specifically, we hypothesize that immunophenotypes prioritizing innate over acquired defenses will be associated with a pattern of accelerated reproductive scheduling, including earlier timing of puberty, age at first sex and age at first birth. Similar associations have been reported across other vertebrate species [27]. From this perspective, the balance of innate versus acquired immunity can be understood as part of the suite of life history traits that co-vary on the fast–slow continuum in relation to extrinsic mortality risk.

In addition, these life history correlations may represent a trade-off between investments in maintenance and reproduction. If reproductive investments are more intensive, then they limit resources available for immunity. As noted earlier, we hypothesize that limited resources will favor investments in innate immunity over acquired immunity, due to the high developmental costs of acquired immune defenses.

Finally, we suggest that in humans, relative resource allocation towards innate versus acquired immune defenses may differ between the sexes. Men across a variety of populations exhibit higher age-specific mortality than women and have a shorter life expectancy [84–86]. Because of their longer life expectancy we hypothesize that women would prioritize investment in acquired immune defenses to a greater degree than men. Existing data fit this pattern as men are known to have higher incidence of infectious diseases than women throughout their lifespan [87, 88] while women are more susceptible to autoimmune diseases [89].

### Preliminary evidence

Relatively few studies have considered measures of both innate and acquired immunity in healthy humans, in community-based settings. However, several studies have reported associations that are consistent with our hypotheses. For example, increased acquired immunity (as indicated by enhanced antibody response to vaccination) has been associated with higher levels of exposure of infectious microbes in infancy [90]. Conversely, infectious exposures in infancy have been negatively associated with inflammation in adulthood [91]. Similarly, several studies have reported negative associations between the prenatal nutritional environment (as measured by birth weight) and inflammation in adulthood [91–93]. Recent studies have also documented positive associations between inflammation and prior maltreatment, harsh family environments and socioeconomic adversity in childhood [94–97], as well as negative associations between childhood adversity and acquired immune function in adulthood [98, 99]. Last, recent analyses document a negative association between levels of inflammation and the antibody response to vaccination within individuals [100], as well as between inflammation and normal levels of IgE [33], findings consistent with the possibility of trade-offs in investments across subsystems of immune defense.

## CONCLUSIONS

Immune defenses provide critical protection against infectious disease, but they require resources to fuel their development and function. We propose that the balance of investment in innate versus acquired immunity is variable and that this balance is optimized in response to signals in the local environment due to the distinct profiles of costs and benefits associated with each type of defense. Nutritional abundance, high pathogen exposure and low signals of extrinsic mortality risk should all favor relatively higher levels of investment in acquired immunity, while undernutrition, low pathogen exposure and high mortality risk favor innate immune defenses.

Our model draws on, and extends, prior research in ecological immunology attempting to explain variation in innate versus acquired immunity. We integrate predictions from the ‘pace of life’ and ‘antigen exposure’ perspectives, recognizing that both sets of factors influence immunophenotypes across a range of species. Furthermore, the majority of studies have centered on relatively small-bodied vertebrates on the opposite end of the ‘live fast and die young’ continuum, as compared with humans. Developmental plasticity is a key mode of adaptation for the human species [74, 101], and we therefore expect that findings from cross-species analyses serve as a reasonable basis for predicting within-species variation in human immunity. On the other hand, in smaller, shorter-lived species life history trade-offs are likely to be more pronounced, as smaller animals have limited energy reserves to balance competing resource demands [102]. Therefore, differences in relative levels of investment in innate versus acquired immune defenses are likely to be smaller in humans, and perhaps more difficult to detect.

Our framework has the potential to help organize prior empirical work documenting the long-term effects of early environments on adult immunity and inflammation in humans. Many chronic degenerative diseases are influenced by experiences during early development [103], and several studies point to aspects of immune function—inflammation in particular—as potentially important physiological pathways connecting nutritional, infectious and psychosocial environments in infancy and childhood with disease risk later in life [91, 92, 94, 96]. These associations have been interpreted primarily in mechanistic terms, with undernutrition acting as a constraint on immune development, and infectious exposures and child maltreatment both influencing the development of regulatory pathways that control inflammation. An evolutionary life history perspective goes beyond mechanism and points to specific aspects of the developmental ecology as likely drivers of the immunophenotype, proposing novel hypotheses regarding the causes and consequences of variation in multiple dimensions of human immunity. In particular, it reconceptualizes nutritional, infectious and psychosocial ‘exposures’ as signals of risk and demand in the local environment that inform patterns of investment in physiological systems.

Furthermore, it questions the assumption that increased levels of investment in innate immunity are inherently pathological. In affluent industrialized settings, higher inflammation—a key component of innate immunity—is consistently associated with increased risk for chronic degenerative diseases [1–3]. However, the fitness costs of morbidity/mortality risk are greatly reduced after the peak reproductive years [104]. Increased investments in innate immunity, earlier in development and during the prime reproductive years, may be adaptive if they increase survival and/or free up resources for other life history processes, like growth or reproduction, even if they increase risk for disease later in life. A broader life history framework encourages a reconceptualization of inflammation as an immune defense that is a component of the organism’s life history strategy, with costs and benefits that are calibrated against the local ecology.

The ‘hygiene hypothesis’ represents an alternative, but not necessarily contradictory, framework for understanding the long-term effects of infectious exposures on immune development and function. Rates of allergy and asthma have increased in affluent industrialized populations at the same time that regimes of sanitation and hygiene have dramatically reduced exposure to infectious microbes. Several studies have connected these recent historical trends, demonstrating that low levels of infectious exposure early in infancy and childhood bias immune development and regulatory processes in ways that increase the likelihood of inflammatory and atopic diseases later in life [105–107]. The hygiene hypothesis emphasizes the role infectious exposures play in guiding the development of immuno-regulatory networks, and suggests that disease emerges as a result of a mismatch between contemporary, overly sanitized environments and the expectations of an immune system that evolved in environments characterized by a greater intensity and diversity of microbial and macroparasite exposure [108, 109].

The framework outlined here goes beyond the hygiene hypothesis in considering nutritional resources and extrinsic mortality risk as additional determinants of immune development. However, the predictions regarding the impact of infectious exposures are similar: Higher levels of infectious exposure early in life should be associated with reduced inflammation later in life. The hygiene hypothesis emphasizes the consequences of mismatch between contemporary and evolutionary environments with respect to the diversity and intensity of microbial exposure, while our framework highlights the potential for developmental plasticity to calibrate investments in immunity to local ecological conditions, within the constraints of available resources and competing life history demands.

Additional research is needed to evaluate the extent to which our framework can explain variation in innate versus acquired immunity in contemporary environments characterized by high levels of obesity, and a paucity of infectious exposures, two situations that are increasingly common but novel in the context of human evolutionary history. Excess adipose tissue contributes to chronic inflammation [110], so one might expect a U-shaped association between nutritional environments and innate immunity, rather than the simple linear association we proposed earlier. However, our framework focuses on the importance of nutritional environments early in life, during sensitive periods of immune development, and prior research has shown that the strength of association between adult overweight/obesity and inflammation is reduced in higher infectious disease environments [111]. It is therefore possible that positive associations between adult adiposity and inflammation reflect, at least in part, the relative absence of microbial exposures in infancy, since obesigenic and highly hygienic environments tend to co-vary. Testing hypotheses from our framework in a wide range of ecological and epidemiological settings will be necessary to determine whether it applies globally, or whether its explanatory power is limited in contemporary environments of affluence.

Testing these hypotheses will, ideally, include multiple measures of acquired and innate immunity. Applying the same measures across populations that differ with respect to nutritional resources, pathogen pressure, and extrinsic mortality risk will be useful for documenting the range of variation in immunity, and for evaluating the utility of our model for explaining this variation. Within-population analyses, preferably with prospectively collected data beginning in infancy, will be necessary to document immune trade-offs in individuals, and to consider the life history and clinical implications of different patterns of investment in innate versus acquired defenses. In addition, attention should be given to the cellular, hormonal and molecular mechanisms that establish and maintain differential patterns of immune investment. Methodological constraints associated with collecting and processing blood samples for assessing immune parameters will pose a challenge to these tests, but minimally invasive options for field-based research are rapidly expanding [112, 113].

An adaptationist, ecological approach to research on human immunity is an important complement to the mechanistic and clinical emphasis of biomedical immunology. Our framework has the potential to make significant contributions to research in human ecological immunology and evolutionary medicine, and our hope is that it will encourage novel insights into the causes and consequences of variation in multiple aspects of human immunity.

**Conflict of interest:** None declared.
